# Serum Angiogenic and Anti-angiogenic Markers in Pregnant Women with Placenta Percreta

**DOI:** 10.4274/balkanmedj.2016.1890

**Published:** 2018-01-20

**Authors:** Hacer Uyanıkoğlu, Adnan İncebıyık, Ahmet B. Turp, Güler Çakmak, Sibel Sak, Neşe G. Hilali

**Affiliations:** 1 Department of Obstetrics and Gynecology, Harran University School of Medicine, Şanlıurfa, Turkey

**Keywords:** Placenta percreta, placental growth factor, fms-Like tyrosine kinase 1, vascular endothelial growth factor

## Abstract

**Background:**

Placenta percreta is the morbidly adherent form of all the placental invasion abnormalities. The pathology that underlies placenta percreta is poorly understood.

**Aims:**

To compare the levels of circulating vascular endothelial growth factor, placental growth factor and soluble fms-like tyrosine kinase 1 in pregnant women with placenta percreta to a control group.

**Study Design:**

Case-control study.

**Methods:**

Twenty-two women who underwent caesarean section due to placenta percreta and 22 women who underwent caesarean section for other obstetric reasons were included in this study. The diagnosis of placenta percreta was defined as extreme trophoblastic invasion involving serosa of the uterus. Venous blood samples were collected for biochemical comparison of circulating vascular endothelial growth factor, placental growth factor and soluble fms-like tyrosine kinase 1 from all pregnant women.

**Results:**

Women with placenta percreta were significantly older, had higher gravidity, received more frequent antenatal steroids and blood transfusions and delivered at an earlier gestational age when compared to the control group. In women with placenta percreta, preoperative circulating levels of vascular endothelial growth factor, placental growth factor and soluble fms-like tyrosine kinase 1 were lower than the controls (p<0.001, p<0.001 and p<0.05, respectively). While the postoperative levels of vascular endothelial growth factorand soluble fms-like tyrosine kinase 1 levels were higher in placenta percreta (p=0.001 and p<0.001, respectively), placental growth factor levels were similar in both groups.

**Conclusion:**

The findings of this study suggest that a decrease in vascular endothelial growth factor, placental growth factor and soluble fms-like tyrosine kinase 1 levels may be related to placenta percreta etiopathogenesis.

Placenta percreta (PP) is the most advanced form of all the placental invasion abnormalities. Although the pathogenesis of PP is not clear, some risk factors are well known, such as previous caesarean section (CS), curettage, uterine surgery, advanced maternal age and multiparity (1). The increased incidence of placenta previa and morbidly adherent placenta (MAP) has been releated to the rising number of previous CS deliveries. Studies estimated that MAP frequency has changed from 3% in women with one CS to 60% in women with 3 previous CSs (2).

Placental vascular growth is necessary to transport oxygen and nutrients to the foetus. Vascularisation of the placental villi starts on the 21^st^ day of conception (3). Certain vasoactive proteins, cytokines, proteases and growth factors contribute to this process. In particular, fine coordination among placental growth factor (PlGF), vascular endothelial growth factor (VEGF) and soluble fms-like tyrosine kinase 1 (sFlt-1) is important for normal placental development and trophoblast invasion (4,5,6). VEGF and PlGF are members of the VEGF family and have synergistic effects in angiogenesis. Soluble endoglin and sFlt-1 irreversibly prevent PlGF and VEGF, affecting their endothelial cell receptors, so that exhibit antiangiogenic effect which leads to endothelial dysfunction (7). Serum sFlt-1 has been shown to increase 50-fold during delivery in a normal pregnant woman compared to a nonpregnant woman (8). Although this placental overexpression of sFlt-1 and its physiological role are not known, it has been speculated that sFlt-1 might prepare the placenta for separation at delivery (6,8). Therefore, we hypothesised that the disturbance of VEGF, PlGF and sFlt-1 production may play a crucial role in PP.

There are numerous studies on the role of PlGF, VEGF and sFlt-1 in patients with preeclampsia, intrauterine growth restriction (IUGR), fetal alcohol syndrome and gestational trophoblastic diseases (GTD) (9,10,11). However there are only a few studies on these parameters in MAP (1,12). In this study, we evaluated how impressed VEGF, PlGF and sFlt-1 levels were in patients with PP and in normal pregnant women both preoperatively and postoperatively.

## MATERIALS AND METHODS

This study was conducted between June 2015 and April 2016 in the Department of Obstetrics and Gynaecology and the Biochemistry Departments of Harran University, Medical Faculty, Şanlıurfa, Turkey. This study conformed to the principles of the 2008 Declaration of Helsinki and was approved by the local ethics committee of Harran University, Medical Faculty, Turkey (Approval number: 15.06.2015/121). Detailed information was given to all pregnant women enrolled in the study, and all the participants signed consent forms.

This study included 22 pregnant women who underwent CS due to PP and 22 pregnant women who underwent CS for preterm labour and previous CS. Women with multiple gestations, cardiovascular and endocrinological disorders, preeclampsia, IUGR and other types of MAP (acreata and increta) were excluded from the study. The levels of PlGF, VEGF and sFlt-1 in both preoperative and postoperative blood of all women were studied. Diagnosis of PP was made by ultrasonographic examination (Voluson 730 scanner, GE Medical systems, Milwaukee, USA) which showed absent or thinning myometrial tissue (less than 1 mm) at the placental site and irregularity or disappearance of the retroplacental echolucent area between the myometrium and the placenta. In addition, increased vascularity of the uterine serosa-bladder interface and vascular invasion of the bladder were observed during Doppler ultrasonography (13). Magnetic resonance imaging (MRI) was not needed for diagnosis in these cases. Preoperative diagnosis of PP was confirmed as extreme trophoblastic invasion that involved the serosa of the uterus during postoperative histopathologic evaluation.

Maternal age, gravidity, parity and gestational age were documented for all pregnant women, and venous blood samples were also collected pre- and postoperatively for biochemical comparison of the groups.

### Blood collection and immunoassay procedures

Venous blood samples were retrieved from all women. Blood collection was performed before the operation and on the first postoperative day. Serum samples were spun at 4000xg for 10 minutes, and the supernatant was extracted and stored at -80 °C until PlGF, VEGF and sFlt-1 levels were measured using specific immunoassays.

VEGF (Cat No: E-EL-H2569, Elabscience) and PlGF (Cat No: E-EL-H1555, Elabscience, China) measurements were in picograms per millilitre (pg/mL). An enzyme-linked immunosorbent assay kit (Cat No: E-EL-H1087, Elabscience) was used in the quantification of sFlt-1 levels in nanograms per millilitre (ng/mL). The other blood analyses (except for biomarkers of angiogenesis), were performed within 2 hours of blood sampling using a haematology analyser (CELL-DYN Ruby, Abbot Laboratories, Abbott Park, Illinois, USA) in the biochemistry laboratory of our hospital.

Until the study ended, the researchers were unaware of the results of the angiogenesis biomarkers in relation to participants’ clinical features including age, gravidity, parity, perinatal morbidity and mortality, number of previous CS and gestational week at delivery.

### Statistical analysis

Statistical Packages for Social Sciences (SPSS) for Windows, Version 20.0 (SPSS, Chicago, IL, USA) was used for statistical analyses. The general linear model was performed for power analysis. When we used a type I error of 0.05 and type II error of 0.20, we found that the power of statistical analysis for preoperative VEGF and PlGF variables was 80% and for preop sFlt-1 variable it was 51%.

All data were expressed as means and standard deviations. Comparisons between preoperative and postoperative values of the groups with normally distributed variables were checked with a dependent sample t-test. The between-group differences without normal distribution were performed using the Mann-Whitney U test, and categorical variables were compared with the Pearson chi-square test. Differences in the groups’ biochemical parameters were analysed using the independent sample t-test. Correlations between VEGF, PlGF and sFlt-1 values and other parameters were assessed by the Pearson’s correlation coefficient. Multivariate linear regression analysis was used to determine the effects of independent variables on dependent variables. Adjustment was performed for confounding factors (age, gravida and parity). A value of p<0.05 was considered significant for statistical analyses.

## RESULT

The demographic features of PP patients and the control group are listed in Table 1. Compared to the control group, women with PP were significantly older, had higher gravidity and parity, more frequently received antenatal steroids and blood transfusions and delivered at an earlier gestational age. As expected, the frequency of bleeding, caesarean hysterectomy and bladder injury was higher in the PP group. The infants of PP women had lower birthweights than those of women with normal pregnancy (2500 vs 2850 g, p&gt;0.05). However, this difference was not statistically significant because the selected control group had the same gestational week (from women with preterm labour and previous CS) as the PP group.

Intraoperative bladder injuries were observed in 2 patients with PP (9.09%), and supra- and infraumbilical median incisions were performed in 19 women (86.4%) in this group. Postoperatively, the PP group also saw 14 cases of bleeding (63%), 1 case of pulmonary embolism (4.5%) and 4 cases of wound infection (18.2%). No intra- and postoperative complications were observed in the control group. Maternal or neonatal mortality was not observed in either group. In the PP group, partial resection and B-Lynch suture were performed in 14 women (63.7%), and a caesarean hysterectomy was performed in 8 women (36.3%) based on treatment type.

**Figure 1:**
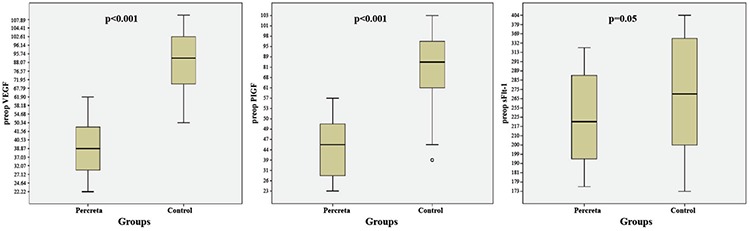
Preoperative circulating levels of vascular endothelial growth factor, placental growth factor and soluble fms-like tyrosine kinase 1 in both groups.

**Figure 2:**
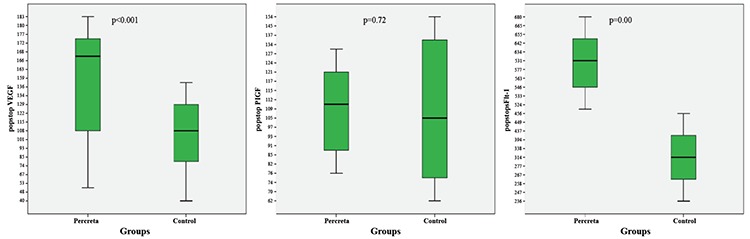
Postoperative circulating levels of vascular endothelial growth factor, placental growth factor and soluble fms-like tyrosine kinase 1 in both groups.

Preoperative circulating levels of VEGF, PlGF and sFlt-1 were lower in the PP group than the control group (p<0.001, p<0.001 and p<0.05, respectively). Although the PP group had higher postoperative levels of VEGF and sFlt-1 (p=0.001 and p<0.001, respectively), the groups had similar PlGF levels (p=0.72) (Table 2). Figures 1 and 2 present the groups’ pre- and postoperative maternal serum concentrations of VEGF, PlGF and sFlt-1. Pearson correlation analysis showed that there was no correlation between VEGF, PlGF and sFlt-1 values and maternal age, gravidity and parity numbers (all p&gt;0.05). Multivariate regression analysis also did not detect any effects of maternal age, gravidity and parity numbers on serum preop VEGF, PlGF and sFlt-1 levels. When we applied an adjustment analysis, we found that age, gravida and parity did not affect the dependent variables (VEGF, PlGF and sFlt-1) (all p&gt;0.05) (Table 3).

## DISCUSSION

The placenta is an organ that provides the transport of oxygen and nutrients from the mother to the foetus in humans (1). A variety of proangiogenic (PlGF and VEGF) and anti-angiogenic factors sFlt1 are increased by the developing placenta, and the balance among these factors is important for normal placentation. Deficient trophoblastic invasion of the maternal tissue can lead to pregnancy complications, such as IUGR and preeclampsia, while excessive invasion can lead to MAP, such as PP (14).

Many studies have addressed the role of PlGF, VEGF, and sFlt-1 in patients with preeclampsia, preterm labour, IUGR, fetal alcohol syndrome, and GTD (6,9,11). Boufettal et al. (9) reported that VEGF expression increases during GTD and might be related to abnormal trophoblastic invasion. Compared to the control group, Semczuk-Sikora et al. (10) found significantly lower maternal serum concentrations of VEGF and PlGF and higher sFlt-1 levels in pregnancies with IUGR. Although some new reports indicated the association between preterm labour and angiogenic factors (sEng, PlGF, PIGF/sEng and VEGFR-1) (15), we selected a control group with the same gestational age as the percreta group to achieve gestational age similarity between the groups.

There are only a few studies evaluating the role of PlGF, VEGF and sFlt-1 in MAP (1,12). We hypothesised that PlGF, VEGF and sFlt-1 serum levels could be important preoperative biomarkers of PP compared to normal fundal placentation. To the best of our knowledge, no study has investigated pre- and postoperative serum VEGF, PlGF and sFlt-1 levels. Therefore, our study is the first to show the role of angiogenic and anti-angiogenic markers in PP in both the preoperative and the postoperative periods.

In early pregnancy, extravillous cytotrophoblast cells leave the placental villus and invade the decidua, myometrium and uterine vessels (16). The maternal decidua, in which the decidual defect is a major contributing factor to PP formation, have a role in the expression of angiogenic growth factors (VEGF and PlGF). It has been suggested that a paracrine mechanism mediates trophoblastic invasion (6). Wehrum et al. (1) found that women with aberrant myometrial invasion had significantly lower serum VEGF levels than the matched control group. Wehrum et al. (1) speculated that the excessive invasion of the myometrium could facilitate the local accrual of VEGF and a significant decrease in the maternal circulatory levels of this angiogenic factor. Although we did not study the local levels of VEGF at the decidual placental interface, we confirmed that circulating maternal VEGF levels in the preoperative blood samples were significantly lower in women with PP than in the control group.

A recent article reported comparable circulatory levels of PlGF, VEGF and sFlt-1 in placental abnormality and control groups (12). This study included 4 groups of women with conditions other than percreta: women with complete placenta previa without MAP, women with accreta and increta, women with percreta and women with normal pregnancy. However, only preoperative blood samples were collected. In our study, we found lower serum levels of VEGF, PlGF and sFlt-1 in the preoperative period. Moreover, postoperative PlGF levels were similar in both groups, but VEGF and sFlt-1 levels were higher in the PP group than the control group. Postoperative increases in VEGF and sFlt-1 concentrations might be associated with the release of these mediators from the invasive placental bed to the peripheral blood after delivery.

Tseng et al. (17) observed increased VEGF expression in patients with placenta accreta. However, it is important to note that Tseng et al. (17) did not evaluate the maternal circulatory levels of VEGF. The authors only documented histopathologic evaluation and did include patients delivered in the second trimester and with some co-morbid disorders. In our study, patients in both groups had no associated co-morbidities and delivered in their third trimesters. Women with confounding factors such as preeclampsia, IUGR and placenta accreta and increta were also been excluded from our study.

sFlt-1 can be considered a mediator that modulates VEGF and PlGF activities (11,18). Recently, several studies demonstrated that serum levels of sFlt-1 increased at an earlier gestational week in women with preeclampsia than the controls (7,8). Additionally, McMahon et al. (19) found lower levels of sFlt-1 protein expression in hysterectomy specimens associated with MAP, suggesting that sFlt-1 plays a critical functional role in the regulation of placental invasion. In the present study, we found that women with PP had significantly lower preoperative sFlt-1 levels and significantly higher postoperative sFlt-1 levels than the control group. Although these results suggest that disruption of sFlt-1 production might play a crucial role in the development of PP, the result is inconclusive because of the low sample size. Thus, it requires more studies with larger samples.

This study has several limitations. First, the study has only maternal serum values of these parameters, not the local levels at the decidual placental interface. Second, the sample size of the study is low because of the rarity of PP. Third, the age and gravidity of the groups were mildly different; however, we did not find any correlation between maternal age and gravidity with PlGF, VEGF and sFlt-1 levels in both correlation and multivariate regression analyses. In conclusion, we demonstrated that life-threatening uteroplacental neovascularisation typical of PP might be correlated with these molecular changes. In pregnant patients with suspected PP, low serum VEGF, PlGF and sFlt-1 levels should alert clinicians to the possibility of PP and to its attendant increase in maternal and fetal morbidity and mortality. Further studies are needed to clarify these responsible signalling molecules in patients with PP.

## Figures and Tables

**Table 1:**
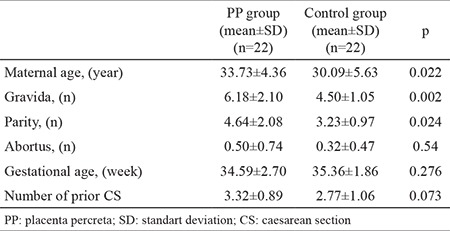
Demographic and clinical characteristics of the PP and control groups

**Table 2:**
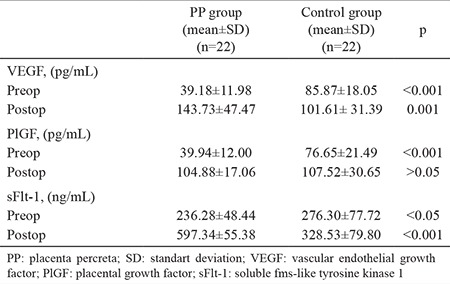
Pre- and postoperative circulating levels of VEGF, PlGF and sFlt-1 in both groups

**Table 3:**
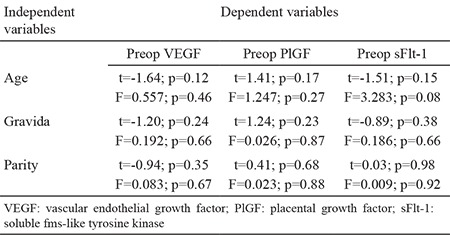
Multiple linear regression analyses of variables
